# Communication of the diagnosis to Klinefelter subjects: an observational study on a key moment of the patient’s life

**DOI:** 10.1007/s40618-024-02302-9

**Published:** 2024-02-20

**Authors:** A. Garolla, M. Kiesswetter, S. Angelini, F. Cavalieri, C. Foresta, M. Panzeri, A. Ferlin

**Affiliations:** 1 Andrology and Reproductive Medicine & Centre for Klinefelter Syndrome, Department of Medicine, University of Padova, Padua, Italy; 2 Institute of Psychology, UMIT TIROL, University of Health Sciences and Technology, Hall in Tirol, Austria; 3 Department of Developmental Psychology and Socialisation, Padua University, Padua, Italy

**Keywords:** Klinefelter syndrome, Time of diagnosis, Communication of the diagnosis, Sexual health, Acceptance of the diagnosis

## Abstract

**Purpose:**

Klinefelter syndrome (KS) is the most prevalent sex chromosome disorder among males. The communication of the KS diagnosis holds significant implications for the diagnosis's acceptance. Recently, the increased use of prenatal diagnostic procedures has raised the question of whether, when, and by whom information, once provided to parents, should be communicated to their children/adolescents. Currently, there is limited information on this topic. This study aims to investigate the most suitable timing, content, and healthcare professionals (HCPs) according to KS patients’ suggestions for conveying the diagnosis, analyzing the impact of communicating the KS diagnosis on patients and their reception of the communication in real-life situations. Furthermore, research entails a comparison of the actual communication and the patients' preferred mode of communication.

**Methods:**

Self-reported interview data was collected from 196 adults diagnosed with KS. The interview was structured, consisting of 32 multiple-choice questions covering various areas related to diagnosis communication.

**Results:**

Most patients with Klinefelter syndrome reported that earlier communication would have been beneficial. Communication before the age of 18 and by parents increased the likelihood of overcoming negative consequences and relying on psychological support.

**Conclusion:**

To mitigate the adverse effects of poorly timed and inadequately delivered communication, typically by a single person, it is advisable that such communication be carried out at the onset of adolescence by an interdisciplinary team of HCPs (including psychologists, geneticists, endocrinologists) and parents. The information provided should not solely concentrate on hormonal and fertility aspects, but also consider other factors such as psychological variables.

**Supplementary Information:**

The online version contains supplementary material available at 10.1007/s40618-024-02302-9.

## Introduction

Klinefelter syndrome (KS) is the most common sex chromosomal disorder in males with a prevalence rate of 1 in 600 individuals. KS commonly leads to hypogonadism and infertility (Ferlin et al., 2018; Lanfranco et al., 2004). Most patients present a 47, XXY karyotype, although mosaicism or the presence of multiple supernumerary X chromosomes have also been detected (3). KS is a common, yet significantly underdiagnosed condition with considerable medical, psychological, and social implications (4). Despite the relatively high incidence of KS, many individuals living with this condition remain undiagnosed (5, 6), due to considerable phenotypic variability, lack of familiarity, and lack of recognition among healthcare providers. It seems that only 25% to 40% of the KF subject pool are ever identified (3, 7, 8). In particular, about 10% would be diagnosed during the prepubertal period while the bulk of patients would be diagnosed during adulthood, typically in the course of a fertility evaluation (9). Only a small proportion would be diagnosed quite late in life, after the age of 50 (8, 9). On the other hand, in recent years, this condition has been increasingly detected through prenatal testing (5). While the main features of KS in childhood are cryptorchidism, hypospadias, speech delay, learning disabilities, gynecomastia, and delayed puberty (10, 11) in adults they are tall stature, gynecomastia, decreased facial hair, hypergonadotropic hypogonadism, small testes, and infertility (7, 9). If these features are present in childhood or adolescence, a general chromosomal panel is usually ordered, which will reveal KS if present. A fundamental moment in the lives of individuals with KS is the communication of the diagnosis, especially in young patients. Inadequate preparation by health care providers could lead to depression, anxiety, or disrupted self-esteem in children, and it is plausible to think that the same could happen in adolescents or adults (4, 12)Men diagnosed with KS later in life complained of late diagnosis and reported similar or more distressing problems than those diagnosed at a younger age, suggesting that they would benefit from early diagnosis and psychological intervention (4, 13). Delayed diagnosis often leaves families searching for answers related to their son's physical and neurodevelopmental concerns for years (14). Parents must then decide when and how to disclose this information to their son. Healthcare professionals (HCPs) are very important partners in this endeavor, but since there are no official recommendations or guidelines on this topic, it can be difficult for them to properly support parents and talk to patients (5). For patients diagnosed after birth, Tremblay and colleagues (2016) propose a personalized approach, whereas Aliberti and colleagues (2022) advocate for a team of professionals, including an endocrinologist, psychologist, geneticist, and parents, to communicate the diagnosis before the age of 18.

An examination of the factors that affect the timing of diagnosis communication would aid in delivering appropriate services to those with KS, their loved ones, and their HCPs (13). Several factors can impact the effects of a chronic disease on patients, including illness severity, social and psychological support, access to national healthcare, treatment efficacy, and community acceptance. This study utilizes a self-administered structured interview to examine the experiences of KS patients with regards to receiving a diagnosis.

## Methods

### Study design

The study was conducted in two phases. The preliminary four-month pilot phase developed the structured interview and evaluated its clarity and completeness allowing for necessary adjustments before the study began. The second phase utilized a cross-sectional design, obtaining self-reported data from men previously diagnosed with KS through a 32-item structured interview alongside sociodemographic information.

### Structured interview

The structured interview was self-reported and anonymous. It included 32 items and respondents answered on a multiple-choice basis using a 5-point Likert scale ranging from "not at all" to "very much," or on a yes/no basis to gather information in the following areas: timing and methods of communication of the diagnosis (e.g. In your opinion, what do you think is the most appropriate time to receive the notification of the diagnosis?), completeness of the information received (e.g. Did you feel the need to seek additional information after being told about your diagnosis?), emotional experiences and fears (Please indicate the extent to which you experienced each of the following moods following the communication of your diagnosis, e.g. fear, anger, anxiety), presence of a supportive network or support groups (e.g. Please indicate the extent to which you are satisfied with the support you have received from family and friends), self-image with the syndrome (e.g. Following the communication of your diagnosis, did your self-image change?), confrontation with the other (e.g. Some people with KS may perceive themselves as "different". If this has happened to you, please indicate how you feel "different" from others.), and Usefulness of psychological support (e.g. Have you ever used psychological support?).

The full English version of the structured interview can be found in the Supplementary Material.

### Procedure

Consecutive patients diagnosed with Klinefelter syndrome (KS) who were seeking endocrine or reproductive consultation were included in the study. The patients were recruited from the Unit of Andrology and Reproductive Medicine at Padua and gave informed consent to participate, having full knowledge of their genetic diagnosis. The andrologists informed subjects about the procedure, providing details about sociodemographic data collection and the structured interview. Participants were instructed to respond to the interviews truthfully. Approval was obtained from the University Hospital of Padua Ethics Committee (Protocol No. 2357P). All participants provided written informed consent before their inclusion. Confidentiality was ensured through the use of a code association with each patient's name. To retrieve the patient's medical history, only the code appeared on the questionnaire. The procedures utilized in this study align with the principles outlined in the Declaration of Helsinki.

### Participants

A total of 196 consecutive KS patients were enrolled in this investigation. Their average age was 37.7 + 10.6 years. Regarding education levels, 1.9% reported completing primary school, 26.2% reported finishing middle school, 54.2% reported completing high school, and 17.8% reported obtaining a university degree. The average age of diagnosis was 25.5 ± 9.7 years. All participant socio-demographic and clinical characteristics are presented in Table 1.

**Table 1:** Socio-demographic and clinical characteristics of the participants

		*M*		*SD*
Age		37.70		10.60
Age of diagnosis communication		25.55		9.72
	N		%	
Education				
Primary school	2		1.90	
Middle school	28		26.20	
High school	58		54.20	
University	19		17.80	
Regular check-ups				
Yes	161		95.80	
No	7		4.20	
Followed by a Specialized centre				
Yes	159		94.60	
No	9		5.40	
Communication by				
HCP	140		85.50	
Parents	20		12.10	
HCP + parents	4		2.40	

### Data analysis

The study data was analyzed using SPPS software and expressed as mean + SD or absolute or percentage frequency. We excluded 28 participants who did not answer all items. To assess content validity, we employed Content Validity Ratio (CVR) and Content Validity Index (CVI). Reliability was assessed through Cronbach’s alpha. Pearson’s correlations were utilized for exploratory assessments of associations, and the Mann–Whitney test and linear regression were employed for inferential assessments. Alpha was set at 0.05 in all analyses.

## Results

The structured interview was evaluated by 15 experts in both endocrinology/andrology and sexology/psychology. The content validity of the interview was excellent (CVI = 0.59): CVR of items ranged from 0.33 to 1 (see Table 1S). We decide to keep all items to have a better understanding of the impact of diagnosis communication. Table 2 presents the correlations between the variable Age and the Anxiety about different aspects, Negative feelings after communication of the diagnosis, and Acceptance of the diagnosis. There was a significant negative correlation between age and Anxiety, Discouragement and Uncertainty after communication, and Desire to hide aspects of the physical appearance. Conversely, age showed a positive significant correlation with Acceptance of the diagnosis. More than half (53.4%) of diagnoses and 72.1% of communications regarding such diagnoses were made during adulthood. According to the survey, 79% of respondents expressed their desire to receive the diagnosis communication earlier, with 41.3% preferring to receive it during pre-adolescence. Only 20.8% stated that they would have preferred to receive it after the age of 18. An andrologist or urologist is the preferred professional to communicate the diagnosis, according to 36.2% of the sample. This was followed by parents at 22.7% and an interdisciplinary team consisting of various specialists at 18.4%. In 73.2% of cases, patients indicated that the person communicating the diagnosis should provide the patient with additional information about the syndrome. The distribution of satisfaction with the method of communication was as follows: 45.8% reported feeling satisfied, 28.6% expressed being relatively satisfied, and 25.6% were not satisfied at all. Among the patients, 50% reported satisfaction with the information provided on the syndrome, while 30.4% were relatively satisfied, and 19.6% felt dissatisfied with the received information. Nearly 74% of the patients stated their desire to seek more information. Internet searches (50.7%) and consulting with another specialist (40.5%) proved to be the primary methods used to obtain further knowledge. The communication of a diagnosis brought about discomfort predominantly associated with infertility (88.1% of cases), metabolic conditions (50.9%), sexual traits (35.1%), and sexuality (31.5%). More than two-thirds of the participants (67.3%) stated that they did not have any prior concerns about their health before being diagnosed. Participants who had any suspicions before diagnosis primarily reported physical health issues. For the majority of participants (66.5%), their self-concept remained unchanged after being diagnosed with KS. Only 19.2% of cases reported difficulty accepting the diagnosis. Additionally, more than one-third of the present sample expressed dissatisfaction with the support provided by family and friends, with nearly 40% indicating that their personal network did not comprehend their condition.

**Table 2:** Pearson correlations between *Age* and Anxiety about different aspects, Negative feelings after communication of the diagnosis, and Acceptance of the diagnosis

	r	*p*
Infertility	− .14	.86
Metabolic diseases	.01	.92
Cardiovascular diseases	.01	.96
Development of sexual characteristics	− .11	.15
Cognitive development	− .01	.88
Development of language	− .09	.27
Psychological disorders	− .06	.46
Sexuality	− .08	.33
Fear after communication	− .22	** < .01**
Sadness after communication	− .01	.86
Discouragement after communication	− .17	**.03**
Disappointment after communication	− .07	.37
Anger after communication	− .12	.13
Feelings of inferiority after communication	− .07	.38
Feelings of humiliation after communication	− .02	.79
Uncertainty after communication	− .17	**.03**
Feelings of impotence after communication	− .04	.58
Feelings of diversity after communication	− .09	.28
Anxiety after communication	− .13	.10
Feelings of shame after communication	.02	.77
Feelings of demotivation after communication	− .10	.21
Changes in the self-image after communication	− .01	.88
Acceptance of the diagnosis	.16	**.04**
Deterioration of the state of health	− .10	.19
Desire to hide aspects of the physical appearance	− .20	**.01**

The correlations between the variable Acceptance of the diagnosis and Anxiety about different aspects, Negative feelings after communication of the diagnosis, and Acceptance of the diagnosis are presented in Table 3. Acceptance of the diagnosis is significantly and negatively correlated with Disappointment, Anger, and Anxiety after communication, Deterioration of the state of health, and Desire to hide aspects of the physical appearance. While the majority of participants stated they were unconcerned about their current condition, a significant proportion (92.2%) emphasized the significance of regular interdisciplinary examinations. Concerning potential health decline, the responses from the sample population were evenly distributed among the various options, without emphasizing either positive or negative extremes. Most respondents (66.7%) reported no need to hide any aspect of their physical appearance. However, in other instances, respondents disclosed concerns associated with the small size of the testicles or penis (36.2%), aspects related to the abdomen and hips (21.3%), and gynecomastia (21.3%). The main areas that caused anxiety in KS patients were about 50.6% of the cases of the ability to procreate, 31.5% of having a normal appearance of sexual characters, and 21.4% of having a normal sexual performance. Thirty percent of survey respondents reported never perceiving any diversity while over thirty-eight percent believed it could be beneficial to engage with others who have the same syndrome. Nearly half of the participants (43.60%) reported having received psychological support at some point in their lives. Among them, 28.90% sought help due to difficulties related to KS. While few participants had psychological support due to KS, 63.70% of them expressed that such support could be beneficial.

**Table 3:** Pearson correlations between Acceptance of the diagnosis and Anxiety about different aspects, and Negative feelings after communication of the diagnosis

	r	*p*
Infertility	− .11	.16
Metabolic diseases	.09	.25
Cardiovascular diseases	.09	.26
Development of sexual characteristics	.09	.26
Cognitive development	.00	1.00
Development of language	.02	.79
Psychological disorders	− .04	.64
Sexuality	.02	.77
Fear after communication	− .13	.10
Sadness after communication	− .09	.23
Discouragement after communication	− .12	.11
Disappointment after communication	− .21	** < .01**
Anger after communication	− .20	** < .01**
Feelings of inferiority after communication	− .12	.14
Feelings of humiliation after communication	− .09	.27
Uncertainty after communication	− .13	.09
Feelings of impotence after communication	− .09	.25
Feelings of diversity after communication	− .12	.13
Anxiety after communication	− .18	**.02**
Feelings of shame after communication	− .07	.37
Feelings of demotivation after communication	− .15	.07
Changes in the self-image after communication	− .00	.97
Deterioration of the state of health	− .22	** < .01**
Desire to hide aspects of the physical appearance	− .26	**.001**

The results of the correlations between the variable Usefulness of psychological support, and Anxiety about different aspects, Negative feelings after communication of the diagnosis, and Acceptance of the diagnosis are presented in Table 4. The correlation between Anxiety about different aspects and Negative feelings after the communication of the diagnosis was significant, with the exception of Acceptance of the diagnosis and Anxiety regarding infertility.

**Table 4:** Pearson correlations between the perception of Psychological support usefulness and Anxiety about different aspects, Negative feelings after communication of the diagnosis, and Acceptance of the diagnosis

	r	*p*
Infertility	.06	.44
Metabolic diseases	.24	** < .01**
Cardiovascular diseases	.31	** < .001**
Development of sexual characteristics	.28	** < .001**
Cognitive development	.31	** < .001**
Development of language	.23	** < .01**
Psychological disorders	.40	** < .001**
Sexuality	.36	** < .001**
Fear after communication	.17	**.03**
Sadness after communication	.22	** < .01**
Discouragement after communication	.22	** < .01**
Disappointment after communication	.22	** < .01**
Anger after communication	.19	**.02**
Feelings of inferiority after communication	.31	** < .001**
Feelings of humiliation after communication	.21	** < .01**
Uncertainty after communication	.31	** < .001**
Feelings of impotence after communication	.33	** < .001**
Feelings of diversity after communication	.26	**.001**
Anxiety after communication	.23	** < .01**
Feelings of shame after communication	.16	** < .05**
Feelings of demotivation after communication	.18	**.02**
Changes in the self-image after communication	.24	** < .01**
Acceptance of the diagnosis	.04	.64
Deterioration of the state of health	.27	** < .001**
Desire to hide aspects of the physical appearance	.27	** < .001**

Alpha for Negative feelings after communication of the diagnosis was 0.93 while for Anxiety about different aspects was 0.87. Negative feelings after communication of the diagnosis (U = 1152.50; p =  < 0.01), Thinking that KS impacts sexual orientation (U = 1269.00; p = 0.02), Health worsening (U = 897.50; p < 0.001), Need to hide physical appearance (U = 537.00; p < 0.001), Usefulness of psychological support (U = 1099.00; p < 0.01), and Anxiety about different aspects (U = 1168.00; p < 0.01) were significantly higher when the communication was before age of 18, while Acceptance of diagnosis (U = 2265.00; p = 0.02) was significantly lower. Negative feelings after communication of the diagnosis (U = 1012.00; p < 0.05), Thinking that KS impacts sexual orientation (U = 988.00; p < 0.01), Health worsening (U = 905.50; p = 0.02), Need to hide physical appearance (U = 824.50; p < 0.01), were significantly higher when communicated by parents.

Table 5 shows the results of the linear regression  analysis of the usefulness of psychological support as dependent variable. The independent variables included Communication before 18 years, Communication by a professional, Negative feelings after communication of the diagnosis, Anxiety about different aspects, Satisfaction about communication, Satisfaction about information received, Thinking that KS impacts on sexual orientation, Changes in self-image, Acceptance of diagnosis, Worries about health, Usefulness of being treated by a specialized equipe, Health worsening, Need to hide physical appearance, Parents and friend support satisfaction, and Close persons comprehension. The effect was significant (F(15,130) = 3.51; p < 0.001). R^2^ = 0.29 showed the model explains 29% of the variance. The variables that significantly increased the perception of the Usefulness of psychological support were Communication before 18 years (β = 0.67; t = 2.96; p = 0.04), Communication by a professional (β = 0.69; t = 2.20; p = 0.03), and Thinking that KS impact on sexual orientation (β = 0.39; t = 3.05; p < 0.01).

**Table 5:** Regression analysis for the prediction of the Usefulness of psychological support according to the scores of Communication before 18 years, Communication by a professional, Negative feelings after communication of the diagnosis, Anxiety about different aspects, Satisfaction about communication, Satisfaction about information received, Thinking that KS impacts on sexual orientation, Changes in self-image, Acceptance of diagnosis, Worries about health, Usefulness of being treated by a specialized equipe, Health worsening, Need to hide physical appearance, Parents and friend support satisfaction, and Close persons comprehension

		β	t	*p*
Communication before 18 years		0.19	2.06	**.04**
Communication by a professional		0.18	2.20	**.03**
Negative feelings after communication of the diagnosis		0.10	0.87	.38
Anxiety about different aspects		0.18	1.89	.06
Satisfaction about communication		− 0.01	− 0.10	.92
Satisfaction about information received		− 0.06	− 0.50	.62
Thinking that KS impacts on sexual orientation		0.25	3.05	** < .01**
Changes in self-image		0.12	1.40	.16
Acceptance of diagnosis		0.09	1.04	.30
Worries about health		− 0.01	− 0.13	.90
Usefulness of being treated by a specialized equipe		0.12	1.45	.15
Health worsening		0.02	0.20	.85
Need to hide physical appearance		0.00	0.02	.99
Parents and friend support satisfaction		0.07	0.76	.45
Close persons comprehension		− 0.11	− 1.25	.21

A linear regression (reported in Table 6), with the dependent variable of Negative feelings after communication of the diagnosis and the independent variables including Communication before 18 years, Communication by a professional, Satisfaction about communication, Satisfaction about information received, Thinking that KS impact on sexual orientation, Changes in self-image, Acceptance of diagnosis, Worries about health, Usefulness of being treated by a specialized equipe, Health worsening, Need to hide physical appearance, Parents and friend support satisfaction, Close persons comprehension, and Psychological support had a significant effect (F(14,129) = 8.01; p < 0.001). R^2^ = 0.47 showed the model explains 47% of the variance. The variables that significatively increased Negative feelings after communication of the diagnosis were Changes in self-image (β = 0.27; t = 3.97; p < 0.001), Worries about health (B = 0.41; t = 4.36; p < 0.001), and Need to hide physical appearance (β = 0.14; t = 2.24; p = 0.03).

**Table 6:** Regression analysis for the prediction of Negative feelings after communication of the diagnosis according to the scores of Communication before 18 years, Communication by a professional, Satisfaction about communication, Satisfaction about information received, Thinking that KS impact on sexual orientation, Changes in self-image, Acceptance of diagnosis, Worries about health, Usefulness of being treated by a specialized equipe, Health worsening, Need to hide physical appearance, Parents and friend support satisfaction, Close persons comprehension, and Psychological support

	β	t	*p*
Communication before 18 years	− 0.05	− 0.58	.56
Communication by a professional	0.04	0.58	.56
Satisfaction about communication	− 0.18	− 1.84	.07
Satisfaction about information received	0.02	0.15	.88
Thinking that KS impact on sexual orientation	− 0.05	− 0.65	.51
Changes in self-image	0.29	3.97	** < .001**
Acceptance of diagnosis	0.06	0.80	.43
Worries about health	0.35	4.36	** < .001**
Usefulness of being treated by a specialized equipe	− 0.11	− 1.59	.11
Health worsening	0.13	1.58	.12
Need to hide physical appearance	0.19	2.24	**.03**
Parents and friend support satisfaction	0.08	1.07	.29
Close persons comprehension	0.03	0.45	.65
Psychological support	0.08	1.19	.24

Linear regression (shown in Table 7) with the dependent variable of Anxiety about different aspects after communication and the independent variables including Communication before or after 18 years, Communication by a professional, Satisfaction about communication, Satisfaction about information received, Thinking that KS impact on sexual orientation, Parents and friend support satisfaction, Close persons comprehension, Changes in self-image, Acceptance of diagnosis, Worries about health, Usefulness of being treated by a specialized equipe, Health worsening, Need to hide physical appearance, and Psychological support was significant (F(14,129 = 3.01; p < 0.001). R^2^ = 0.28 depicted the model explains 28% of the variance. The variables significantly increased Anxiety about different aspects were Worries about health (β = 0.23; t = 2.23; p = 0.03), and Health worsening (β = 0.19; t = 2.69; p < 0.01).

**Table 7:** Regression analysis for the prediction of Anxiety about different aspects after communication according to the scores of Communication before or after 18 years, Communication by a professional, Satisfaction about communication, Satisfaction about information received, Thinking that KS impact on sexual orientation, Parents and friend support satisfaction, Close persons comprehension, Changes in self-image, Acceptance of diagnosis, Worries about health, Usefulness of being treated by a specialized equipe, Health worsening, Need to hide physical appearance, and Psychological support

	β	t	*p*
Communication before or after 18 years	− 0.03	− 0.31	.75
Communication by a professional	0.05	0.62	.54
Satisfaction about communication	− 0.01	− 0.06	.95
Satisfaction about information received	− 0.08	− 0.71	.48
Thinking that KS impact on sexual orientation	0.06	0.73	.46
Parents and friend support satisfaction	0.13	1.53	.13
Close persons comprehension	− 0.06	− 0.73	.47
Changes in self-image	− 0.04	− 0.48	.63
Acceptance of diagnosis	0.10	1.21	.23
Worries about health	0.21	2.23	**.03**
Usefulness of being treated by a specialized equipe	0.03	0.36	.72
Health worsening	0.25	2.69	**.01**
Need to hide physical appearance	0.17	1.72	.09
Psychological support	0.14	1.72	.09

## Discussion

This study generated results and knowledge about the opinions in different areas of patients with KS. In this survey patients with Klinefelter's syndrome were diagnosed on average at the age of 25 years which is in line with another study (9). This previous research showed that the majority of diagnoses are made in adulthood, although there have been more and more premature diagnoses in recent years (5). The communication of the diagnosis is often delayed due to many parents feeling unable to share the information with their child: this conflicts with the desires of patients, who would prefer to receive such diagnostic communication earlier in life, during adolescence or even pre-adolescence.

Our findings indicate that when the diagnosis is disclosed by parents before the age of 18, there is a greater likelihood of reporting the usefulness of psychological support. However, such disclosure also results in an increase in negative emotions, concerns about potential health deterioration, a desire to conceal one's appearance, and fears that KF may impact sexual orientation. This data is easily explained by the fact that sexual orientation strengthens with age. It can be assumed that older participants have a stable sexual orientation and therefore do not question it. Our data highlighted that negative emotions following the diagnosis disclosure are amplified by alterations in self-perception, concerns about current health and the need to conceal one's appearance. We are aware that younger individuals place a greater emphasis on self-image, whereas older individuals tend to prioritize other personality and factual aspects, which weaken the importance of self-image (16). This phenomenon may shed light on the findings regarding concerns about actual health. It is possible that older individuals are more confident in their health status due to their longer experience with the syndrome. Additionally, younger individuals are less inclined to stereotype and dislike psychotherapy, instead they are more likely to believe in its potential to assist them (17). When considering communication from parents, it is important to note that the parents in our sample were not adequately prepared, if at all, for this important task. The distress experienced by parents can potentially have negative effects on the child, as parents themselves may experience fear (18).

Furthermore, it should be noted that if the young patients have not been extensively prepared for this fundamental moment of the communication of the KS diagnoses, this may lead to a child’s depression, anxiety, or disrupted self-esteem (12). Therefore, it is plausible to think that the same happens for adults. Moreover, in the case of prenatal diagnoses and diagnoses in early childhood, the parents should be extensively accompanied and educated. This is also because about a quarter of the patients interviewed stated that they would like to receive the diagnoses from their parents, while more than two-thirds of their parents were not able to communicate it to them. Even if the diagnosis is given by a professional, a very important point is the completeness of the information that the patients receive, because a very high percentage said that the person who gives them the diagnosis should give them a further explanation. This is underlined by the fact that only half of the patients were satisfied with the information they received, and they had to seek further information i.e., on the web or by asking another specialist. Therefore, patients must understand the diagnosis and be accompanied in this process to counteract fears and disappointments since this is related to the acceptance of the diagnosis. Although the acceptance of the diagnosis does not correlate with the perception of psychological support usefulness, a large proportion of participants reported that they would have liked psychological support, but only a small proportion of participants received it. Our study found that only concerns about KF potentially affecting sexual orientation, communication of the diagnosis before the age of 18, and by parents had a significant impact on perceptions of the usefulness of psychological support. The significant connections between these perceptions and the adverse emotions and concerns about the physical and psychological effects that emerge after diagnosis emphasize the importance of providing psychological support to patients when necessary. Hence, the literature suggests a tailored approach (5). If there is currently a lack of consensus on the most appropriate timing for communicating a diagnosis to boys with KF syndrome, it is advisable to discuss the diagnosis and treatment with girls who have Turner syndrome as soon as they are capable of understanding the information (Frías & Davenport 2003; Tremblay et al. 2016). Based on these recommendations and research findings, it is advisable that a comprehensive team comprising endocrinologists, psychologists, geneticists, and parents carry out the diagnosis during or shortly before adolescence, as proposed by Aliberti (2022). To our knowledge, there is a lack of research on the psychological impact of communicating the diagnosis in adulthood, but we can imagine that even if earlier communication may lead to more struggle about possible negative consequences, younger patients may also be more inclined than older ones to seek or accept psychological support to face their possible fears, anxieties or doubts and to find an effective way of dealing with KF. A further consideration is that testosterone replacement therapy or sperm banking may be beneficial for certain patients beginning in late puberty (19, 20). This may also reduce the main concern of adult patients, namely sterility problems (13).

The study produced relevant data on the impact of communicating a Klinefelter Syndrome diagnosis and patient preferences in this regard. Practitioners may derive some practical implications from these findings. Various studies have demonstrated that psychoeducation is a valuable tool for reducing stress and anxiety. (21). Classical stress management training techniques, including emotional regulation, cognitive restructuring, and relaxation techniques, may be applicable in this context (22). Assertiveness training or support groups can assist individuals in managing similar situations. Support groups offer the added benefit of reducing the fear of negative social evaluation, creating an optimal environment for discussing personal difficulties, fears, and needs.

One limitation of this study is its cross-sectional design. A longitudinal approach would provide a better understanding of patients' feelings and distress. Furthermore, we did not evaluate depression, anxiety, or other psychological symptoms through validated questionnaires. Additional clinical and demographic information, such as socioeconomic status and religiosity, would enhance the interpretability of the results. Furthermore, due to the fact that the data collection was limited to Padua, Italy, the generalizability of the findings to other contexts may be limited. This study lacks the potential insights that KF participants could provide through qualitative research methods such as focus groups or semi-structured interviews. It would be important discuss primary concerns regarding the communication of the diagnosis with adults who have KF, and also with parents of minors with KF, and the involved professionals.

## Conclusions

In conclusion, this study presents evidence regarding the impact of communicating a KS diagnosis to patients. Additionally, various negative (psychosocial) aspects were identified which can be moderated through a personalized approach. To alleviate fear and stress related to the impact of communication, psychological interventions could potentially be useful in reducing the burden and enhancing social inclusion.

### Supplementary Information

Below is the link to the electronic supplementary material.

Supplementary file1 (DOCX 42 KB)

## Data Availability

The datasets generated during and/or analysed during the current study are available in the Research Data Unipd repository, https://researchdata.cab.unipd.it/id/eprint/972,
