# Supplementary material for: Communication of the diagnosis to Klinefelter subjects: an observational study on a key moment of the patient’s life
Source: J Endocrinol Invest. 2024 Feb 20;47(8):2029–39. doi: 10.1007/s40618-024-02302-9 (PMC11266383; doi:10.1007/s40618-024-02302-9)
Supplement: Supplementary file 1 — Supplementary file1 (DOCX 42 KB) [file 40618_2024_2302_MOESM1_ESM.docx]

Supplementary Information

Translation of the structured interview

Date:_____ Age:______ Region of residence:____________

If foreign country specify which one:_______________

Educational qualification:

- None
- Elementary school
- Middle school
- High school
- University degree

Please provide below the name of the city in which you were notified of your diagnosis of Klinefelter Syndrome: __________________

Please indicate below the name of the center where you were notified of the diagnosis (e.g., department, hospital, institution, ...): _________________

Do you receive regular checkups for your syndrome? YES NO

If yes, are you followed by a specialized center for Klinefelter Syndrome? YES NO

1. When were you diagnosed with the syndrome?
   - I don’t know
   - Prenatal age
   - At birth
   - Age: ______
2. At what age did you have the communication of the diagnosis? Age:_____
3. In your opinion, what do you think is the most appropriate time to receive the notification of the diagnosis?
   - Between 5 and 10 years
   - Between 11 and 12 years
   - Between 13 and 14 years
   - Between 15 and 16 years
   - Between 17 and 18 years
   - After the age of 18
   - It would be better not to know
4. By whom do you obtain the communication of the diagnosis?
   - Mother
   - Father
   - Both parents
   - Pediatrics
   - Infant Neuropsychiatrist
   - Gynecologist
   - Psychologist
   - Geneticist
   - Pediatric endocrinologist
   - Adult endocrinologist
   - Andrologist/Urologist
   - Primary Care Physician
   - Other (specify): ______________
5. In your opinion, who should communicate the diagnosis?
   - Mother
   - Father
   - Both parents
   - Pediatrics
   - Infant Neuropsychiatrist
   - Psychologist
   - Geneticist
   - Endocrinologist
   - Andrologist/Urologist
   - Primary Care Physician
   - A multidisciplinary team made up of all these figures
   - Other (specify): ______________
6. Did the person who communicated the diagnosis to you defer the explanation of the syndrome to a different person? If so, which one?
   - No
   - Pediatrics
   - Infant Neuropsychiatrist
   - Gynecologist
   - Psychologist
   - Geneticist
   - Pediatric endocrinologist
   - Adult endocrinologist
   - Andrologist/Urologist
   - Primary Care Physician
   - Other (specify): ______________
7. Would you have wanted a different person to explain the syndrome? If so, which one?
   - No
   - Pediatrics
   - Infant Neuropsychiatrist
   - Gynecologist
   - Psychologist
   - Geneticist
   - Pediatric endocrinologist
   - Adult endocrinologist
   - Andrologist/Urologist
   - Primary Care Physician
   - A multidisciplinary team made up of all these figures
   - Other (specify): ______________
8. Overall, how satisfied are you with how your diagnosis was communicated to you? (time spent, willingness to listen, etc.).

| Not at all | A little | Somewhat | Very | Very much |
| --- | --- | --- | --- | --- |
|  |  |  |  |  |

1. Overall, how satisfied are you with the comprehensiveness of the information you received about the syndrome? (clarity, completeness, etc.)

| Not at all | A little | Somewhat | Very | Very much |
| --- | --- | --- | --- | --- |
|  |  |  |  |  |

1. Did you feel the need to seek additional information after being told about your diagnosis? (More than one answer is possible)
   - No, I did not feel this type of need.
   - Yes, because the communication of the diagnosis received was unclear
   - Yes, because I felt the need to have a different opinion
   - Yes, because I felt the need to explore some aspects of the condition in more detail
   - Other (please specify):______________
2. How did you research additional information? (You can indicate multiple answers)
   - Consulting a specialist
   - Researching on the Internet
   - Consulting scientific journals
   - Consulting a medical encyclopedia
   - Consulting texts
   - Asking relatives/friends/acquaintances
   - Talking to someone with the same condition
   - Contacting an association of patients with SK
   - Other (specify):_____________
3. Who have you decided to talk to about the syndrome among family, friends, and acquaintances? (It is possible to indicate more than one answer)
   - Spouse/Partner
   - Close relatives (parents/brothers)
   - Most relatives (and family members)
   - Family friends
   - My friends
   - Colleagues
   - Wider friends
   - None
   - Other (specify):___________
4. With whom did you decide NOT to talk about it? (You can indicate more than one answer)
   - Spouse/partner
   - Close relatives (parents/brothers)
   - Most relatives (extended family members)
   - Family friends
   - My friends
   - Colleagues
   - Acquaintances
   - None
   - Other (specify):_________
5. Why did you decide not to talk to them about it? (You can indicate more than one answer)
   - Avoiding prejudice
   - Avoiding discrimination
   - To avoid misunderstandings
   - Not to give worries
   - Because I feel ashamed
   - Because I don't think it is necessary
   - Other (please specify):____________
6. When you were notified of your diagnosis, did you receive information about possible discomfort related to the following spheres? (You may indicate more than one answer)
   - Infertility
   - Metabolic diseases (obesity, diabetes, osteoporosis)
   - Cardiovascular diseases (hypertension, heart problems, thrombosis)
   - Development of sexual characteristics (development of mammary glands, reduced hairiness)
   - Cognitive development (attention, learning)
   - Problems in language development
   - Psychological disorders
   - Sexuality
   - None of the above
7. Indicate the extent to which the possibility of developing discomfort for each of the following spheres has caused you concern.

|  |  | Not at all | A little | Somewhat | Very | Very much |
| --- | --- | --- | --- | --- | --- | --- |
| Infertility |  |  |  |  |  |  |
| Metabolic diseases |  |  |  |  |  |  |
| Cardiovascular diseases |  |  |  |  |  |  |
| Development of sexual characteristics |  |  |  |  |  |  |
| Cognitive development |  |  |  |  |  |  |
| Problems in language development |  |  |  |  |  |  |
| Psychological disorders |  |  |  |  |  |  |
| Sexuality |  |  |  |  |  |  |

1. Please indicate the extent to which you experienced each of the following moods following the communication of your diagnosis.

|  | Not at all | A little | Somewhat | Very | Very much |
| --- | --- | --- | --- | --- | --- |
| Fear |  |  |  |  |  |
| Sadness |  |  |  |  |  |
| Discouragement |  |  |  |  |  |
| Disappointment |  |  |  |  |  |
| Anger |  |  |  |  |  |
| Inferiority |  |  |  |  |  |
| Humiliation |  |  |  |  |  |
| Uncertainty |  |  |  |  |  |
| Helplessness |  |  |  |  |  |
| Diversity |  |  |  |  |  |
| Anxiety |  |  |  |  |  |
| Shame |  |  |  |  |  |
| Demotivation |  |  |  |  |  |
| Other (specify) |  |  |  |  |  |

1. At the time you were notified of your diagnosis, did you think the syndrome might affect your sexual orientation? (Heterosexual, homosexual)

| Not at all | A little | Somewhat | Very | Very much |
| --- | --- | --- | --- | --- |
|  |  |  |  |  |

1. Before you learned of your diagnosis, did you ever have any suspicion about your health?
   - Yes
   - No
   - If yes, please specify with respect to what:___________

|  | Not at all | A little | Somewhat | Very | Very much |
| --- | --- | --- | --- | --- | --- |
| 1. Following the communication of your diagnosis, did your self-image change? |  |  |  |  |  |
| 1. Do you feel that you have accepted the diagnosis within yourself? |  |  |  |  |  |
| 1. How concerned are you about your current condition? |  |  |  |  |  |
| 1. How useful do you think it is to be followed periodically by a specialized team? |  |  |  |  |  |
| 1. How concerned are you about a possible deterioration in your health? |  |  |  |  |  |
| 1. Do you feel, or have you ever felt, the need to disguise or hide anything about your outward appearance? |  |  |  |  |  |

If yes, which one? (please specify)

1. Some people with SK may perceive themselves as "different". If this has happened to you, please indicate how you feel "different" from others. (You may give more than one answer)
   - Ability to procreate
   - Psychological balance
   - Physical appearance
   - Sexual performance
   - Intellectual capacity
   - Communication skills
   - Physical health
   - I have never perceived myself as different from others
   - Other (specify):_____________
2. Do you think having a comparison with people with the same disease is helpful? (You can indicate more than one answer)
   - Yes, because this allows you to find full understanding and acceptance.
   - Yes, because it is heartening to know that you are not the only person with a given condition, and helps you not feel alone.
   - Yes, because I can have a role model
   - Yes, because I can have a role model and gain support from it
   - No, I don't think it is useful
   - Other (please specify):______________
3. Have you ever used psychological support?
   - Yes, I requested it myself
   - Yes, it has been recommended to me
   - Yes, it was suggested to me
   - Yes, it was suggested to me
   - I have never felt the need for it
   - Other (specify):__________
4. How useful do you think is it, or can be, to take advantage of psychological support?

| Not at all | A little | Somewhat | Very | Very much |
| --- | --- | --- | --- | --- |
|  |  |  |  |  |

1. If you have received psychological support, was this required because you experienced discomfort concerning Klinefelter's Syndrome?
   - YES
   - NO
2. Please indicate the extent to which you are satisfied with the support you have received from family and friends.

| Not at all | A little | Somewhat | Very | Very much |
| --- | --- | --- | --- | --- |
|  |  |  |  |  |

1. Please indicate the extent to which you believe people close to you can understand your medical condition.

| Not at all | A little | Somewhat | Very | Very much |
| --- | --- | --- | --- | --- |
|  |  |  |  |  |

Any patient comments:

**Table 1S.** The judgment of the 32 items of each of the 15 experts

| Item | Exp1 | Exp2 | | Exp3 | | Exp4 | | Exp5 | | Exp6 | | Exp7 | | Exp8 | | Exp9 | | | Exp10 | Exp11 | | | Exp12 | | Exp13 | Exp14 | Exp15 | Ne | CVR | |
| --- | --- | --- | --- | --- | --- | --- | --- | --- | --- | --- | --- | --- | --- | --- | --- | --- | --- | --- | --- | --- | --- | --- | --- | --- | --- | --- | --- | --- | --- | --- |
| 1 | 1 | 1 | | 1 | | 1 | | 0 | | 1 | | 1 | | 1 | | 1 | | | 1 | 1 | | | 1 | | 1 | 1 | 1 | 14 | .87 | |
| 2 | 1 | 1 | | 1 | | 1 | | 1 | | 1 | | 1 | | 1 | | 1 | | | 1 | 1 | | | 1 | | 1 | 1 | 1 | 15 | 1 | |
| 3 | 1 | 1 | | 1 | | 1 | | 1 | | 1 | | 1 | | 0 | | 1 | | | 1 | 1 | | | 1 | | 1 | 1 | 0 | 13 | .73 | |
| 4 | 1 | 1 | | 1 | | 1 | | 1 | | 0 | | 1 | | 0 | | 1 | | | 1 | 1 | | | 1 | | 1 | 1 | 1 | 13 | .73 | |
| 5 | 1 | 1 | | 1 | | 1 | | 1 | | 0 | | 0 | | 0 | | 1 | | | 1 | 1 | | | 1 | | 1 | 1 | 0 | 11 | .47 | |
| 6 | 1 | 1 | | 1 | | 1 | | 1 | | 1 | | 1 | | 1 | | 0 | | | 1 | 1 | | | 1 | | 1 | 0 | 1 | 13 | .73 | |
| 7 | 1 | 1 | | 1 | | 1 | | 1 | | 1 | | 1 | | 1 | | 1 | | | 1 | 1 | | | 1 | | 1 | 0 | 1 | 14 | .87 | |
| 8 | 1 | 1 | | 1 | | 1 | | 1 | | 1 | | 1 | | 1 | | - | | | 1 | 1 | | | 1 | | 1 | 1 | 1 | 14 | 1 | |
| 9 | 1 | 1 | | 1 | | 1 | | 1 | | 1 | | 1 | | 1 | | 1 | | | 1 | 1 | | | 1 | | 1 | 1 | 1 | 15 | 1 | |
| 10 | 0 | 1 | | 1 | | 1 | | 1 | | 1 | | 1 | | 0 | | 1 | | | 1 | 1 | | | 1 | | 1 | 1 | 1 | 13 | .73 | |
| 11 | 0 | 1 | | 1 | | 1 | | 1 | | 1 | | 1 | | 1 | | 1 | | | 1 | 1 | | | 1 | | 1 | 0 | 1 | 13 | .73 | |
| 12 | 1 | 1 | | 1 | | 1 | | 0 | | 1 | | 0 | | 1 | | 1 | | | 1 | 1 | | | 1 | | 1 | 1 | 1 | 13 | .73 | |
| 13 | 0 | 1 | | 1 | | 1 | | 1 | | 1 | | 0 | | 1 | | 1 | | | 1 | 0 | | | 1 | | 1 | 1 | 1 | 12 | .60 | |
| 14 | 0 | 1 | | 1 | | 1 | | 1 | | 1 | | 0 | | 0 | | 1 | | | 1 | 1 | | | 1 | | 1 | 1 | 1 | 12 | . 60 | |
| 15 | 1 | 1 | | 1 | | 1 | | 1 | | 1 | | 1 | | 1 | | 1 | | | 1 | 1 | | | 1 | | 1 | 1 | 1 | 15 | 1 | |
| 16 | 1 | 1 | | 1 | | 1 | | 1 | | 1 | | 1 | | 1 | | 1 | | | 1 | 1 | | | 1 | | 1 | 1 | 1 | 15 | 1 | |
| 17 | 1 | 1 | | 1 | | 1 | | 1 | | 1 | | 1 | | 1 | | 1 | | | 1 | 1 | | | 1 | | 1 | 1 | 1 | 15 | 1 | |
| 18 | 0 | 1 | | 1 | | 1 | | 0 | | 0 | | 1 | | 0 | | 0 | | | 1 | 1 | | | 1 | | 1 | 1 | 1 | 10 | .33 | |
| 19 | 1 | 1 | | 1 | | 1 | | 1 | | 1 | | 1 | | 1 | | 0 | | | 1 | 1 | | | 1 | | 1 | 0 | 1 | 13 | .73 | |
| 20 | 0 | | 1 | | 1 | | 1 | | 1 | | 1 | | 1 | | 1 | | 1 | 1 | | | 1 | 1 | | 1 | | 1 | 1 | 14 | .87 |  |
| 21 | 1 | | 1 | | 1 | | 1 | | 1 | | 1 | | 1 | | 1 | | 1 | 1 | | | 1 | 1 | | 1 | | 1 | 1 | 15 | 1 |  |
| 22 | 1 | | 1 | | 1 | | 1 | | 1 | | 1 | | 1 | | 1 | | 1 | 1 | | | 1 | 1 | | 1 | | 1 | 1 | 15 | 1 |  |
| 23 | 0 | | 1 | | 1 | | 1 | | 1 | | 1 | | 1 | | 1 | | 1 | 1 | | | 1 | 1 | | 1 | | 0 | 0 | 12 | . 60 |  |
| 24 | 0 | | 1 | | 1 | | 1 | | 1 | | 1 | | 1 | | 1 | | 0 | 1 | | | 1 | 1 | | 1 | | 1 | 1 | 13 | .73 |  |
| 25 | 0 | | 1 | | 1 | | 1 | | 1 | | 1 | | 1 | | 1 | | 1 | 1 | | | 1 | 1 | | 1 | | 1 | 1 | 15 | .77 |  |
| 26 | 0 | | 1 | | 1 | | 1 | | 0 | | 1 | | 0 | | 1 | | 1 | 1 | | | 1 | 1 | | 1 | | 1 | 1 | 12 | . 60 |  |
| 27 | 1 | | 1 | | 1 | | 1 | | 1 | | 1 | | 1 | | 1 | | 1 | 1 | | | 1 | 1 | | 1 | | 1 | 1 | 15 | 1 |  |
| 28 | 1 | | 1 | | 1 | | 1 | | 0 | | 1 | | 1 | | 1 | | 1 | 1 | | | 1 | 1 | | 1 | | 0 | 1 | 13 | .73 |  |
| 29 | 0 | | 1 | | 1 | | 1 | | 1 | | 1 | | 1 | | 1 | | 1 | 1 | | | 1 | 1 | | 1 | | 1 | 1 | 14 | .87 |  |
| 30 | 1 | | 1 | | 1 | | 1 | | 1 | | 1 | | 0 | | 1 | | 0 | 1 | | | 1 | 1 | | 1 | | 0 | 1 | 12 | . 60 |  |
| 31 | 1 | | 1 | | 1 | | 1 | | 1 | | 1 | | 1 | | 1 | | 1 | 1 | | | 1 | 1 | | 1 | | 0 | 1 | 14 | .87 |  |
| 32 | 1 | | 1 | | 1 | | 1 | | 1 | | 1 | | 1 | | 1 | | 1 | 1 | | | 1 | 1 | | 1 | | 0 | 1 | 14 | .87 |  |
| CVI |  | |  | |  | |  | |  | |  | |  | |  | |  |  | | |  |  | |  | |  |  |  | .87 |  |

Note: “0” = experts who judged item as no essential; “1” = experts who judged item as essential; Ne = total number of experts who judged each item as essential; CVR = Content Validity Ratio, calculated as $\frac{Ne-\frac{N}{2}}{\frac{N}{2}}$ , where N is the sample size of experts (15); CVI = Content Validity Index, average of CVR scores.
